# From Translation to Causality: A Two-Axis Map of RNA-of-Origin and Output Attribution in Translated Non-Coding RNAs During Skeletal Muscle Myogenesis and Regeneration

**DOI:** 10.3390/cells15211981

**Published:** 2026-10-30

**Authors:** Yueying Zhang, Keying Li, Xixi Yang, Wei Zhang, Mingtao Ma, Nanjian Luo

**Affiliations:** Department of Histology and Embryology, School of Preclinical Medicine, Zunyi Medical University, Zunyi 563000, China; zhangyueying@zmu.edu.cn (Y.Z.); likeying8890@zmu.edu.cn (K.L.); yangxixiyxx@zmu.edu.cn (X.Y.); zhangwei2396@zmu.edu.cn (W.Z.)

**Keywords:** microprotein, translated non-coding RNA, circular RNA, RNA-of-origin, causal inference, skeletal muscle regeneration

## Abstract

**Highlights:**

**What are the main findings?**

- The T × F framework separates translation/source evidence from output-specific functional attribution, preventing product detection from being conflated with causality.
- LINC00961–SPAR and MTLN meet the qualified, endpoint-specific T4/F4 criteria for defined regenerative phenotypes, whereas the core circRNA cases remain limited by source and/or output resolution.

**What are the implications of the main findings?**

- Translated ncRNA assignments should remain specific to the biological state, model, and phenotypic endpoint rather than being treated as fixed properties of a locus.
- The next decisive experiment should target the unresolved inference: source resolution, translation-selective testing with measured parent-RNA retention, or endpoint-specific causal separation.

**Abstract:**

Transcripts annotated as non-coding can yield both an RNA molecule and a translated product, complicating causal attribution in skeletal muscle. Three distinct claims require different evidence: a product exists, the nominated RNA generated it, and the product drives a defined phenotype. We examine them in myogenesis and regeneration using a qualitative, two-axis evidence map. The T axis records translation and RNA-of-origin evidence, whereas the F axis records experiments separating RNA-dependent from product-dependent effects for a stated model and endpoint. Endogenous coding-selective experiments support qualified product-dependent assignments for LINC00961–SPAR and MTLN/MPM. Linc-RAM/MRLN demonstrates biologically active RNA and peptide outputs, but not that both cause the same phenotype. Among the selected circRNA systems, circZNF609, circFAM188B, circNEB, and circSIK2 retain unresolved source and/or functional attribution, and none meets both T4 and F4. Rather than ranking study quality or assuming transfer across species and myogenic stages, the map identifies the experiment still needed to establish causality.

## 1. Introduction

Skeletal-muscle regeneration proceeds through satellite-cell activation, proliferation, differentiation, fusion, and fibre rebuilding [1,2,3,4]. Classic isolation and lineage studies established regenerative competence and self-renewal [5,6,7,8,9]. Work on fibroblast interactions, the requirement for Pax7-positive satellite cells, and the fusogens Myomaker and Myomixer/Minion/Myomerger further shows that these stages and cellular dependencies are biologically distinct [10,11,12,13,14,15,16]. For translated ncRNAs, this matters because a perturbation may alter one stage but not another; "muscle function" is therefore too coarse an endpoint for causal attribution. We use skeletal muscle as an analytical setting, not because translated ncRNAs are known to be uniquely enriched or preferentially translated in this tissue. Rather, the field offers stage-resolved endpoints of myogenesis and regeneration together with published designs ranging from endogenous output-selective genetics to studies of circular RNAs (circRNAs) in which product provenance or output attribution remains unresolved.

The attribution problem arises from the dual-output potential of a single transcript. Long non-coding RNAs (lncRNAs) can act through sequence, structure, localisation, and macromolecular interactions [17,18], with established skeletal-muscle examples including linc-MD1 [19], MUNC [20], and Dum [21]. When the same transcript also contains a translated open reading frame (ORF), transcript-level gain- or loss-of-function changes both the RNA molecule and its encoded product. A resulting phenotype therefore belongs to the transcript system as a whole and, on its own, does not distinguish an RNA-intrinsic from a product-dependent mechanism.

The distinction between coding and non-coding annotation has become less clear. Small and non-canonical ORFs can generate bioactive products [22,23,24,25]; peptidomics has detected short-ORF products in human cells and tissues [26,27]; and ribosome profiling and comparative analyses have expanded candidate ORFs and supported functional noncanonical proteins [28,29,30,31,32,33,34]. Muscle examples such as DWORF and MRLN illustrate the biological relevance of such reclassification [35,36], while selected circRNAs can undergo cap-independent or N6-methyladenosine (m6A)-associated translation [37,38]. Such findings establish coding capacity, but not which molecular output causes a phenotype. In this Review, “microprotein” is used as the preferred general term for short-ORF-encoded protein products, whereas “micropeptide” is retained where it reflects established terminology for a specific product or the original literature. The two terms are not treated as distinct evidence categories or separated by a fixed size threshold.

Three claims need to be kept separate: the product exists, the nominated RNA generated it, and that product causally mediates the specified phenotype. ORF prediction, ribosome engagement, and endogenous product detection address the first claim to different degrees, but none alone establishes functional causality. Conversely, parent-RNA knockdown or overexpression does not establish an RNA-intrinsic mechanism because multiple outputs are altered together [39]. For circRNAs, source attribution adds another prerequisite because linear or aberrantly spliced RNAs from the same locus or expression construct can generate overlapping products [40,41]. Evidence for one claim cannot substitute for another.

Existing experimental guidance already addresses several parts of this attribution problem. Guidance for lncRNA perturbation [39] and circRNA validation [40,41] specifies controls for transcript identity, experimental expression, and alternative linear templates. Muscle-focused reviews describe non-coding RNA functions, micropeptide mechanisms, and translated ncRNA candidates [42,43,44]. A recent cancer-focused review explicitly organised evidence from coding architecture and ribosome engagement through endogenous peptide detection to functional attribution [45]. Neither these assays nor the need to separate RNA effects from encoded-product effects is new. The T × F map places source-resolved translation evidence alongside endpoint-specific functional attribution, keeping discordant evidence states visible where a single graded sequence might obscure them.

**Figure 1:**
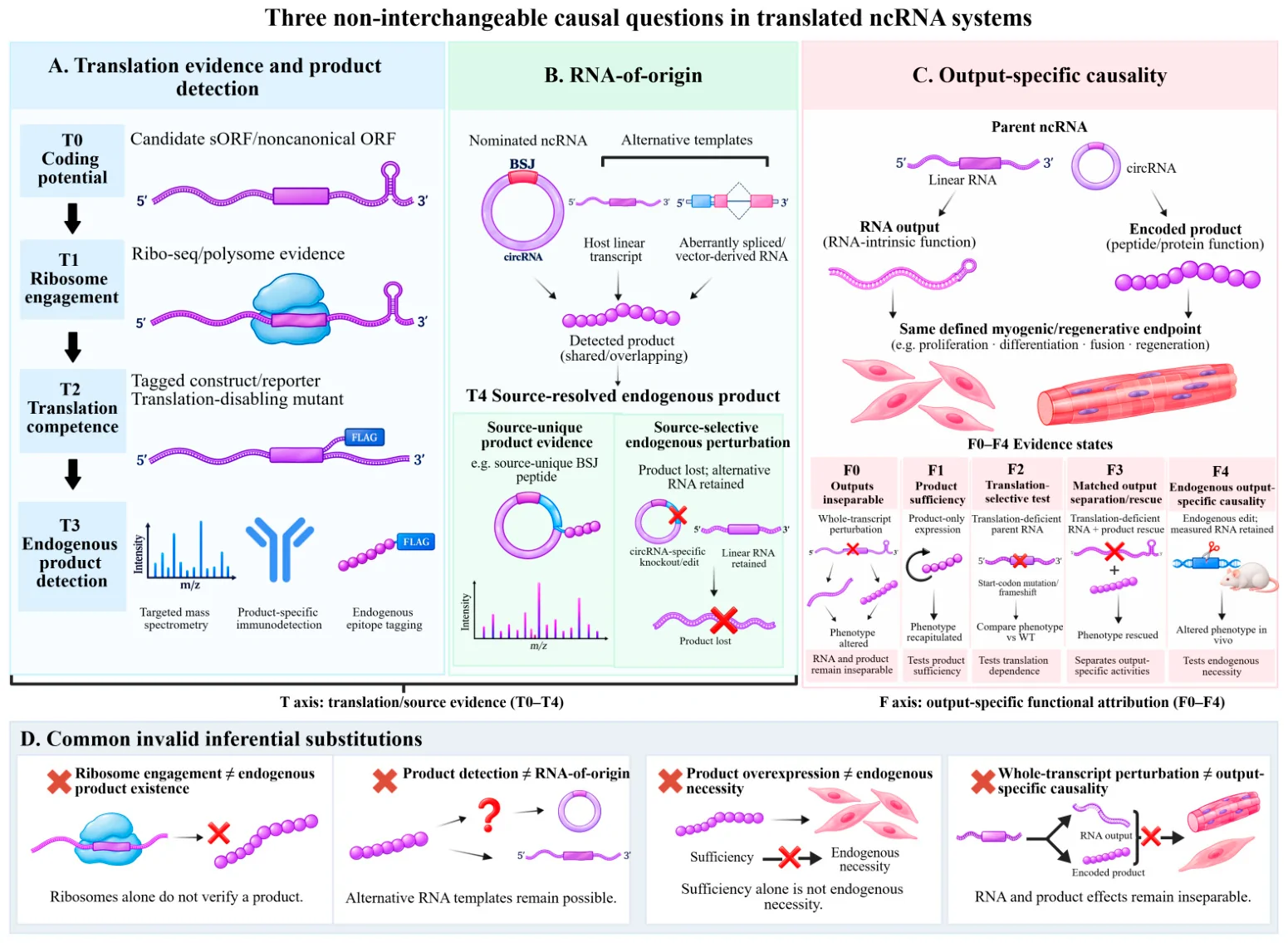
Three non-interchangeable questions for translated ncRNA systems. (**A**) T0–T3 distinguish coding-potential prediction, ribosome engagement, construct translation, and endogenous product detection; only T3 establishes endogenous existence. (**B**) T4 additionally resolves RNA-of-origin against plausible alternative templates (for example, a source-unique BSJ peptide). (**C**) F0–F4 distinguish inseparable outputs, product sufficiency, translation-selective comparison, matched output separation/rescue, and endogenous necessity in vivo. F2 outcomes may persist or attenuate; F4 RNA preservation covers only measured properties. F3 and F4 are noncumulative. (**D**) Four invalid substitutions between product detection, provenance, and causality. BSJ, back-splice junction; ORF, open reading frame. Created with BioGDP.com [46].

The two-axis T × F map is a qualitative aid to interpretation, not a study-quality score or validated ranking instrument. It distinguishes strong product detection with unresolved causal output (for example, circNEB) from translation-selective functional comparisons with incomplete circular-template source resolution (for example, circSIK2). The R/P/B/U labels indicate the supported RNA or product activity within each model, cellular state, and endpoint. The framework does not assume statistical independence of the axes or independent experiments: a well-controlled endogenous perturbation can inform both. The aim is to make the remaining inferential gap and next discriminating experiment explicit, not to claim priority for existing verification standards [39,40,41,45]. The three causal questions and their evidentiary boundaries are summarised in Figure 1.

## 2. Scope and Analytical Framework

This narrative review examines a purposive selection of translation-associated ncRNA systems relevant to skeletal-muscle myogenesis and regeneration. The initial literature search was conducted on 1 October 2026 using PubMed, Web of Science Core Collection, and Scopus, with a publication cutoff of 30 September 2026. During revision, the authors recovered the original search strategies and associated records to complete the methodological documentation. A targeted database-coverage check on 21 October 2026 supplemented the original searches with minor refinements for named RNA/product systems and relevant experimental methods. The case-level evidence matrix and the operational protocol are presented in Supplementary Table S1 and Supplementary Note S1, respectively; Supplementary Note S2 reports the consolidated database-specific search expressions.

Core examples were selected at the level of an experimentally studied RNA/encoded-product system, rather than by individual citation. The seven core systems were selected purposively because they combined at least construct-level translation evidence (T2 or higher), a specified muscle-cell or skeletal-muscle phenotype, and a design relevant to distinguishing product existence, RNA-of-origin, or output-specific causality. The selected linear benchmarks are Linc-RAM/MRLN, LINC00961–SPAR, and MTLN/MPM/LEMP; the circRNA examples are circZNF609, circFAM188B, circNEB, and circSIK2. Studies reporting coding potential without adequate endogenous or functional separation, or examining mechanisms in other systems, were discussed as boundary, emerging or methodological examples where informative, rather than given unsupported core coordinates. Foundational primary papers, subsequent reassessments, and experimental standards were also identified by citation tracing [39,40,41]. This selection compares types of inference; it neither catalogues every translated ncRNA nor estimates how frequently each T/F state occurs.

Available records from the initial search and supplementary coverage check were reviewed and deduplicated by DOI, with normalised titles used when DOI was absent; records with distinct DOIs, including correction notices, were retained separately. Primary studies were checked for experimental claims that determined an assignment. Supplementary Table S2 contrasts the present map with prior approaches, while Supplementary Table S3 lists the names of representative RNA systems and encoded products. The review has no prospective systematic-review protocol or exhaustive PRISMA-style screening. The T × F assignments were independently assessed against predefined operational criteria, with interpretive differences resolved through discussion and consensus; no numerical agreement coefficient is reported. Database-search yields are not interpreted as the number of eligible or included studies.

## 3. A Two-Axis Causal Evidence Framework for RNA and Encoded-Product Functions

Describing a translated ncRNA as biologically bifunctional can conflate three questions: Does an endogenous product exist, which RNA template generated it, and which molecular output causes a defined phenotype? The T axis distinguishes translation competence and endogenous product detection (T0–T3) from source-resolved detection (T4). The F axis instead records evidence that separates a parent RNA’s phenotype from effects of its encoded product. These are distinct inferential questions, not experimentally independent variables: one informative endogenous coding edit may support both source attribution and output-specific necessity. T4 combines endogenous product evidence with source attribution; it is not a prescribed experimental sequence. For example, junction-specific ribosome evidence without a validated endogenous product is reported as supplementary template information at the appropriate lower T state, not automatically as T4. T and F states are qualitative categories, neither equally spaced numerical scores nor mandatory cumulative stages. For every assignment, the model and biological state of the T evidence, the model and endpoint of the F evidence, the decisive experiment, and any untested RNA properties must be specified; cross-context evidence is explicitly labelled and must not be mistaken for a same-context causal demonstration.

### 3.1. Axis I: Translation Source Evidence

T0 denotes computational ORF or coding-potential evidence. PhyloCSF, CPAT, and CPC2 provide complementary ways to nominate coding regions [47,48,49], but prediction alone establishes neither ribosome engagement nor product existence. T1 denotes ribosome engagement compatible with active translation, including Ribo-seq or polysome evidence. Ribosome profiling can reveal translation outside established annotations [50,51,52,53], and ORF-level confidence is strengthened by features such as ribosome-release behaviour and triplet periodicity [54,55]. T1 nevertheless remains evidence of translation-associated ribosome occupancy rather than proof that a stable endogenous product accumulates.

T2 denotes experimentally demonstrated translation competence, for example a tagged or reporter construct, an in vitro translation assay, or a coding-dependent signal lost after a translation-disabling edit. The expected product should be verified in the context of the expression system; for circRNA vectors, linear or aberrantly spliced by-products must be considered. T2 establishes product generation under the tested conditions, but neither endogenous accumulation nor origin specifically from the intended circular template [37,38,40,56,57].

T3 requires direct product evidence in an endogenous setting, such as appropriately controlled immunodetection, endogenous epitope tagging, or mass spectrometry in unmanipulated cells or tissues. Product-identity controls, peptide specificity, and orthogonal assays determine confidence in that observation [31,58,59,60]. A shared peptide or antibody band may support endogenous existence without identifying the RNA template. When endogenous evidence is sparse, challenged, or not replicated, the assignment should remain ‘reported/provisional T3’ rather than be treated as definitive or raised to T4. circSIK2 illustrates this distinction: muscle LC-MS/MS and antibody detection support T3, but do not uniquely establish the circular template [61].

T4 requires both T3-level endogenous product evidence and informative attribution to the specified RNA source, with plausible alternative templates evaluated. For linear systems, disappearance of the endogenous product after a coding-selective locus edit, accompanied by assessment of the host RNA and alternative coding sources, may resolve the source at the gene-locus level; it does not automatically resolve contributions of different RNA isoforms. SPAR and MTLN provide such locus-level examples [62,63]. For circRNAs, a confidently assigned endogenous peptide uniquely traversing a circularisation-dependent junction, with relevant alternative sources excluded, or selective disappearance of the endogenous product after circRNA-specific perturbation while linear host transcripts remain intact, would be informative [40,41,64,65,66,67]. RNase R resistance, divergent PCR, construct-derived translation, and shared host-protein peptides alone do not reach T4. The circNEB junction-associated spectrum reported after SKP1 co-immunoprecipitation is relevant evidence, but it does not resolve provenance without source-uniqueness validation [68].

### 3.2. Axis II: Functional Attribution

Detecting a genuine translated product does not establish that it causes the phenotype attributed to its parent RNA. F0 denotes a phenotype following intact-RNA or whole-locus perturbation when RNA-dependent and translation-dependent effects cannot be separated. If no corresponding phenotype has been measured, F is not assigned rather than presumed F0.

F1 requires an independently expressed encoded product to reproduce a specified functional phenotype or a clearly defined product-level molecular readout under an interpretable control. Expression alone, co-detection alongside an RNA phenotype, and downstream pathway rescue do not meet this criterion. F1 establishes sufficiency in the tested system, but not endogenous translation necessity or independent activity of the parent RNA. circFAM188B-103aa reproduces the direction of proliferation/differentiation changes seen after parent-circRNA expression but does not establish whether the parent-circle phenotype requires translation [69].

F2 requires a matched test of translation-competent and translation-deficient versions of the parent RNA in the same model and for the same endpoint. Loss of the intended product and the measured preservation of the parent RNA must be documented. The functional comparison determines how F2 is interpreted: attenuation after translation loss supports a product contribution, whereas retention of the RNA-associated phenotype despite verified translation loss supports a translation-independent RNA arm. Neither outcome establishes complete molecular equivalence or excludes alternative RNA effects. For circRNAs, circularisation and abundance of the BSJ-defined RNA and expression of the linear host transcript should be assessed; localisation, stability, structure, or RNA-binding properties merit additional testing when relevant [39,40]. A non-significant RNA-abundance difference is not a formal equivalence test. Linc-RAM/MRLN exemplifies persistence of the myogenic RNA effect [70], whereas the circSIK2 ATG-to-ACG expression-construct comparison supports a translation contribution in cultured myoblasts [61].

F3 requires matched output separation for the same phenotypic endpoint, with translation-deficient parent-RNA testing complemented by independent product-only restoration in the deficient background or equivalently controlled reciprocal output-specific comparisons. Restoring a downstream pathway is not equivalent to product-specific rescue; independent product gain-of-function in another model or endpoint is not matched output separation. The MyolncR4/LEMP experiments demonstrate translation-selective loss in C2C12 cells and separate product sufficiency, but no fully matched product-restoration test of the deficient parent-RNA phenotype was reported [71]. For differentiation, these results support F2 rather than a completed F3 assignment.

F4 follows a separate, non-cumulative route: an endogenous coding-selective perturbation must abolish or substantially reduce the target product, retain the parent RNA to the extent directly assessed, and change a defined in vivo phenotype in the corresponding physiological or injury context. A whole-locus or coding-region-plus-3′UTR deletion that removes the RNA does not meet this requirement. F4 does not require the ectopic product-rescue design that defines F3, but the absence of such rescue remains a limitation. Endogenous SPAR start-site editing and the one-base-pair MPM coding-frame insertion satisfy the operational F4 criteria for specified muscle-regeneration endpoints, with host-RNA abundance measured [62,63]. Neither experiment establishes that every RNA property remained unchanged. These are qualified, endpoint-specific product-dependent assignments under the operational F4 criteria, not proof that all parent-RNA properties or alternative cis effects have been excluded.

### 3.3. Four Interpretive Evidence States

T and F assignments lead to four interpretive evidence states, none of which should be treated as a fixed identity of a locus. RNA-intrinsic (R) denotes a defined phenotype that persists when translation is disabled, supporting activity of the RNA output. Product-dependent (P) denotes a phenotype that changes when the encoded product is selectively disrupted while the parent RNA is sufficiently preserved, with product rescue or sufficiency adding support. Experimentally bifunctional (B) denotes a transcript system in which independent activities of both outputs have been demonstrated. System-level bifunctionality means that the RNA and encoded product each show biological activity, even if those activities are measured in different phenotypes or biological readouts. This differs from same-phenotype dual-output causality, in which both outputs make separable causal contributions to one defined phenotype. Linc-RAM/MRLN is therefore a B system because translation-deficient Linc-RAM retains pro-myogenic RNA activity whereas MRLN independently regulates SERCA-dependent Ca^2+^ handling [36,70]. Unresolved (U) denotes systems in which translation or biological activity is established but output-specific causality remains incomplete. No core system reviewed here receives an unambiguous R-only designation under these strict criteria; R is retained as a logically necessary evidence state and as a descriptor of separable RNA arms within bifunctional systems. All assignments remain provisional and endpoint-specific. The absence of an R-only core case should not be generalised to skeletal-muscle lncRNAs as a class. R-only assignments are considered only within the translated-ncRNA core set defined in Section 2; classic RNA-function lncRNAs that do not meet the Review’s translation-evidence entry criterion fall outside this classification rather than constituting negative R cases.

### 3.4. circRNA-Specific Evidentiary Requirements

For circRNAs, establishing that an RNA is circular does not establish that a detected protein was translated from that circle. Apparent translation can arise from linear or aberrantly spliced RNAs generated from the same locus or expression construct. The original circZNF609 study linked the circular transcript to heavy polysomes, translation competence, and myoblast proliferation [72], whereas later systematic analysis showed that proteins attributed to overexpressed circZNF609 constructs could also be produced by linear transcripts [41]. RNase R resistance, divergent PCR, and back-splice-junction sequencing help establish circular-RNA identity [40,64,65,73,74,75], but they do not establish protein provenance.

Attributing a circRNA phenotype to its protein requires three distinct lines of evidence: confirmation of the circular transcript and assessment of linear by-products; endogenous product identification with circular-template source discrimination; and output-selective functional testing for the same defined endpoint. RNase R resistance or a sequenced back-splice junction identifies an RNA species, whereas a peptide traversing the predicted junction is informative for provenance only if its sequence and endogenous origin are independently supported and alternative linear sources are considered. Mutations targeting start codons, IRES-like elements, or m6A sites also require RNA-preservation checks. No single assay necessarily establishes all three inferences [40,41].

### 3.5. A Minimal Experimental Standard for Claiming Bifunctionality

Bifunctionality requires evidence of activity for both the RNA and its encoded product; parallel phenotypes after intact-RNA and peptide expression are not enough. An informative matched design tests the wild-type parent RNA, a translation-deficient parent RNA with explicit preservation checks, and an independently expressed product for a specified model and endpoint. When feasible, reciprocal product- and RNA-selective rescue gives stronger evidence of dual-output causality for the same phenotype. Product-only constructs should avoid grossly supraphysiological expression, and residual activity of a translation-deficient RNA must not automatically be interpreted as an autonomous RNA function [39,40].

Linc-RAM illustrates this logic because a full-length transcript carrying an MRLN-disrupting frameshift retains myogenic activity, directly supporting an RNA-intrinsic arm [70]. Outside the regeneration-focused core, circTmeff1 provides a complementary proof of principle: translation-deficient circTmeff1 retains part of its pro-atrophic activity whereas TMEFF1-339aa is independently active [76]. These examples support transcript-system bifunctionality, but a stronger same-phenotype dual-output claim would require evidence that both outputs make separable causal contributions to the same endpoint.

Assignments are made through a defined sequence of checks. First, specify species, model, biological state, intervention, and phenotypic endpoint. Second, assign T using directly observed endogenous product evidence and source discrimination, recording the context in which each was obtained. Third, assign F from an experiment addressing that same endpoint; distinguish intact-RNA manipulation, product-only sufficiency, translation-selective comparisons (including preserved as well as attenuated phenotypes), matched output rescue, and endogenous in vivo necessity. Fourth, record RNA properties measured, plausible alternative templates, controls, and subsequent contradictory evidence. Without the required discriminator, an assignment cannot be raised; cross-context or provisional evidence is noted explicitly. The case-level rationale is documented in Supplementary Table S1.

Table 1 summarises the evidence required for each category and the limits of what it establishes. F2, F3, and F4 identify different experimental routes; the numerical labels do not denote equal intervals or a compulsory sequence of procedures.

## 4. Linear Transcripts as Causal Benchmarks

### 4.1. Linc-RAM/MRLN: Separable Outputs Without Same-Phenotype Dual Causality

Linc-RAM/MRLN serves here as a benchmark for separable outputs rather than as proof that two outputs co-cause one regeneration phenotype. MRLN is a 46-amino-acid micropeptide encoded by a transcript originally treated as a putative long non-coding RNA and regulates skeletal-muscle Ca^2+^ handling [36]. Subsequent work showed that the same transcript system also has an RNA-dependent role in myogenic differentiation [70]. For the present framework, the key point is experimental separation of the two outputs, not the history of the transcript’s annotation.

The most informative RNA-output experiment disrupted the MRLN reading frame while leaving full-length Linc-RAM intact. Whole-transcript gain- and loss-of-function experiments initially linked Linc-RAM to C2C12 differentiation but could not distinguish RNA-mediated activity from translation of MRLN. Yu et al. introduced a frameshift in the MRLN ORF of full-length Linc-RAM; the translation-deficient RNA retained differentiation-promoting activity similar to wild-type Linc-RAM [70]. This F2-type translation-selective comparison retains, rather than attenuates, the phenotype. It supports an RNA-dependent myogenic arm and must not be misread as evidence that MRLN translation is necessary for differentiation.

Mechanistic data support the RNA arm as well. Linc-RAM associates with MyoD and facilitates assembly of a MyoD-Baf60c-Brg1 transcriptional complex, promoting chromatin engagement and activation of myogenic programmes including MyoG and miR-206 [70]. A cytoplasmic fraction was later reported to interact with muscle glycogen phosphorylase (PYGM), enhance its enzymatic activity, and contribute to myogenic differentiation [77]. These mechanisms are not needed to prove output separation, but they support biological activity of full-length Linc-RAM beyond its role as a translation template.

**Figure 2:**
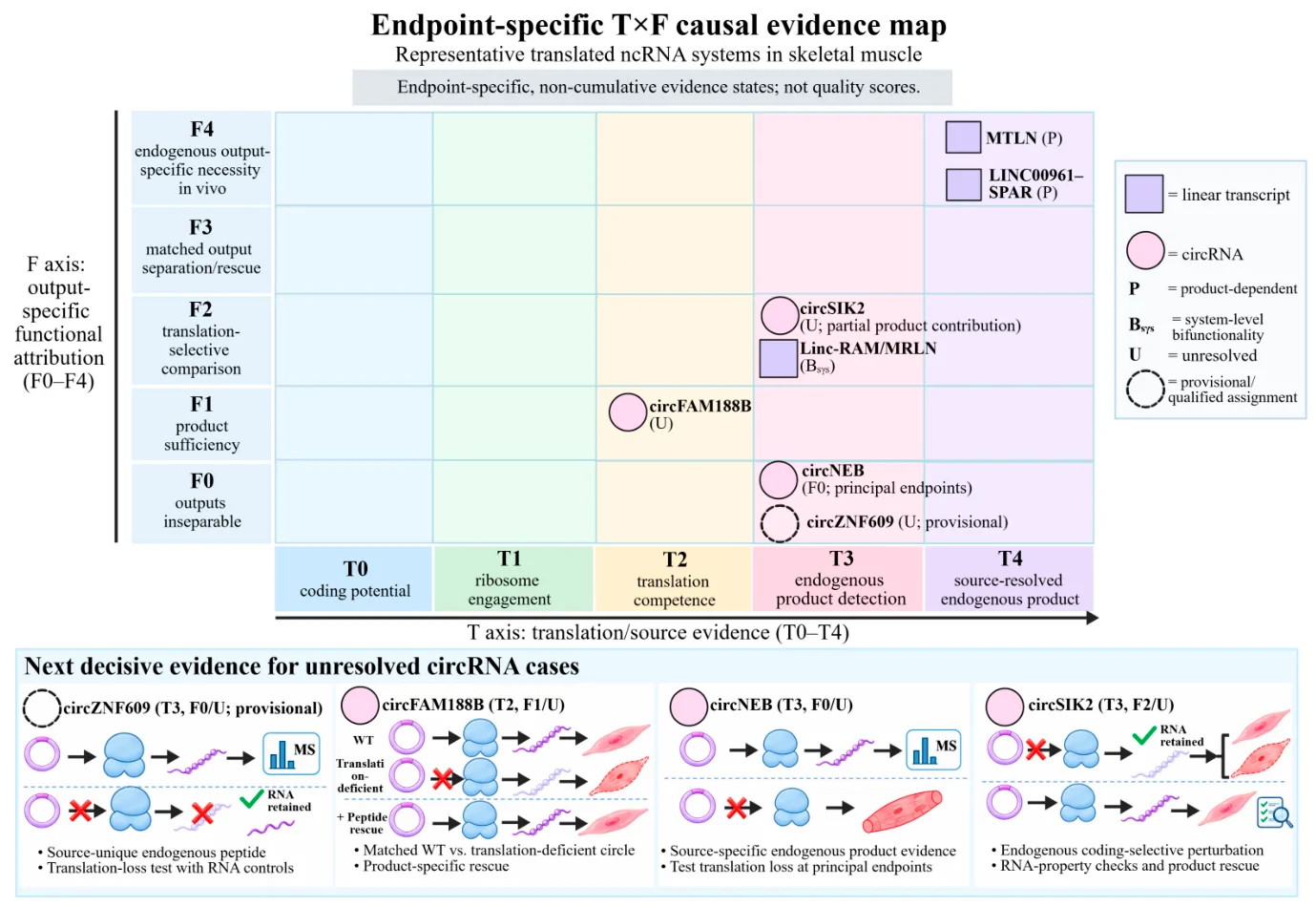
Qualitative T × F map of representative translated ncRNA systems in skeletal muscle. T denotes translation/source evidence; F denotes endpoint-specific output attribution. These are not numerical quality scores or cumulative ranks, and the axes are not experimentally independent. Squares, linear transcripts; circles, circRNAs; dashed outlines, provisional assignments; P, product-dependent; Bsys, system-level bifunctionality at different endpoints; U, unresolved. SPAR and MTLN reach T4/F4/P for specified in vivo phenotypes, with RNA preservation limited to properties measured. Linc-RAM/MRLN (T3/F2/Bsys) does not establish dual causality for the same phenotype. circFAM188B is T2/F1/U; circSIK2, T3/F2/U; circNEB, T3/F0/U for principal myogenic/regenerative phenotypes; circZNF609, provisional T3/F0/U with T and F evidence from different models. Narrow circNEB molecular results do not establish product dependence of those principal phenotypes. The lower panels show proposed next experiments. Created with BioGDP.com [46].

The in vivo genetics support an RNA-dependent contribution to regenerative myogenesis but do not fully resolve both outputs. In the Linc-RAM knockout model, exon 2 was deleted while exons 1 and 3 generated a fusion transcript retaining the MRLN ORF [70]. After cardiotoxin injury, mutant mice showed impaired regeneration and primary myogenic cells differentiated more slowly. Retention of the ORF supports an RNA-based interpretation, but an intact coding sequence does not show that MRLN protein abundance, localisation, and activity were unchanged. The in vivo result is therefore supportive rather than a fully output-specific F4 experiment. An Author Correction published in 2025 replaced representative KO images in Fig. 2e that had been inadvertently duplicated from WT samples; the authors stated that this presentation error did not alter the study conclusions [70].

Independent physiological work establishes the protein arm. MRLN localises to the sarcoplasmic reticulum, interacts with sarco/endoplasmic reticulum Ca2+-ATPase (SERCA), and inhibits Ca^2+^ uptake; genetic loss enhances Ca^2+^ handling and exercise performance [36]. Linc-RAM/MRLN therefore supports a B designation at the transcript-system level, but not same-phenotype dual-output causality. The most defensible conclusion is that one transcript system generates two independently active outputs whose causal contributions must be specified endpoint by endpoint.

### 4.2. LINC00961-SPAR: A T4/F4 Product-Dependent Benchmark in Skeletal Muscle Regeneration

LINC00961 provides a contrasting, strongly supported example of a polypeptide-dependent skeletal-muscle regeneration phenotype. Matsumoto et al. [62] identified a short ORF encoding the small regulatory polypeptide of amino acid response (SPAR, also termed SPAAR). LINC00961 associated with polysomes, tagged constructs produced the predicted polypeptide, disruption of the initiation codon abolished production, and endogenous SPAR-derived peptides were detected by tandem mass spectrometry. These observations establish endogenous product existence; the endogenous SPAR-specific allele described below also supports locus-level source attribution. Together, the evidence places the system at T4 in the present framework.

SPAR localises predominantly to late endosomal and lysosomal membranes and interacts with vacuolar ATPase machinery involved in amino-acid sensing upstream of mTORC1 [62]. Translation-competent LINC00961 suppressed amino-acid-induced mTORC1 signalling, whereas initiation-deficient constructs did not reproduce this effect. The signalling phenotype therefore depends on SPAR production rather than on increased abundance of the host transcript alone.

The decisive in vivo experiment selectively disrupted the endogenous protein output. Rather than deleting the host transcript, Matsumoto et al. disrupted SPAR production by CRISPR/Cas9 while retaining measurable LINC00961 expression. After acute skeletal-muscle injury, SPAR loss enhanced mTORC1 signalling and accelerated aspects of regeneration, including recovery of regenerated fibre size and muscle mass [62]. Dietary leucine deficiency suppressed mTORC1 activity and diminished the SPAR-loss-associated regenerative advantage; this dietary intervention is distinct from pharmacological mTORC1 inhibition. This endogenous, RNA-abundance-qualified design contributes to T4 source attribution and meets the operational F4 criterion for the defined regeneration phenotype.

The product-dependent (P) assignment remains specific to the phenotype considered. In endothelial cells, translation-deficient LINC00961 was later shown to inhibit network formation, whereas the SPAR/SPAAR coding sequence alone had an opposing pro-angiogenic effect, with distinct interaction partners for the two outputs [78]. For skeletal-muscle regeneration, however, LINC00961-SPAR is best placed at T4/F4 and classified as P. The designation does not imply that LINC00961 is biologically inert as an RNA in other tissues or phenotypes.

### 4.3. MTLN: Convergent T4/F4 Evidence for a Product-Dependent System

MTLN has been reported under several names following independent discovery and historical annotation. A 56-amino-acid microprotein encoded by the 1500011K16Rik/LINC00116 locus was independently reported as LEMP [71], MPM [63], MOXI [79], and mitoregulin [80]; MTLN is used here as the canonical term. Independent studies detected the endogenous product and localised it to mitochondria using complementary immunodetection or endogenous-tagging approaches [63,79,80], establishing at least T3 before source-specific genetics are considered.

Cell-based studies offer complementary, although experimentally distinct, evidence for translation-dependent MTLN function. Wang et al. [71] selectively disrupted the LEMP reading frame in C2C12 cells and observed impaired differentiation while measured MyolncR4 RNA abundance remained comparable. The initiation-deficient LEMP construct retained RNA expression but did not reproduce the differentiation-promoting effect. These support a qualified cell-level F2 assignment, supplemented by product-sufficiency evidence, rather than F3, because complete matched product rescue was not reported. The LEMP knockout mice in that study lacked the entire coding region and 3′UTR, with loss of host RNA; those animal data therefore do not independently meet F4 [71].

Lin et al. [63] provided the key in vivo output-selective test using an endogenous MPM allele. A one-base-pair coding-frame insertion introduces premature termination after the fifth amino acid, eliminates the detectable full-length product, and leaves measured skeletal-muscle transcript abundance comparable to wild type. Mutant mice exhibited smaller fibres and impaired physical performance; following cardiotoxin injury, they showed altered myogenic responses and smaller regenerating fibres. These results meet the operational, RNA-abundance-qualified F4 criterion for those endpoints and provide T4-level attribution to the edited locus. Two reported transcript isoforms share the coding region; the experiment does not independently establish which isoform contributes most of the endogenous product. Unmeasured RNA structure, localisation, and interactions remain possible alternatives rather than proven to be unchanged.

Mechanistic studies link the same product to fatty-acid beta-oxidation, respiratory supercomplexes, respiration, lipid metabolism, and membrane-associated regulation [63,79,80,81,82], although submitochondrial localisation and proposed partners are not fully uniform across models. This heterogeneity does not determine the causal classification. For the differentiation and regeneration endpoints considered here, the P assignment rests on translation-selective genetics, especially the endogenous coding-frame mutation that removes product while preserving transcript abundance, rather than on any single proposed molecular partner. MTLN is therefore T4/F4 and product-dependent for the defined phenotypes, while possible RNA-intrinsic functions in other contexts remain open.

### 4.4. Unresolved Linear Transcripts: Translation Evidence Without Causal Separation

The chicken lncRNA-Six1 study is an adjacent example rather than a core skeletal-myogenic case. Cai et al. identified seven candidate ORFs within lncRNA-Six1 and found that ORF2 could generate an approximately 7.26-kDa product in an expression-based assay [83]. The principal lncRNA-Six1- and ORF2-directed proliferation, migration, and promoter-activity experiments were performed in DF-1 cells rather than a skeletal-myogenic model, although primary myoblasts were used for localisation-related observations. The study therefore does not meet the strict core-case criterion used here. It nevertheless illustrates an important evidentiary pattern: translation competence and product sufficiency can be demonstrated without establishing endogenous product necessity or an independent RNA function. We therefore discuss lncRNA-Six1 as an adjacent T2/F1-like example in its tested cellular context, without assigning it a core R/P/B/U classification.

Recent translatome-scale studies are accumulating candidate products faster than causal experiments can resolve them. By integrating RNA-seq and Ribo-seq during differentiation of goat skeletal muscle satellite cells, Pan, Lou, Jing, et al. [84] identified 25 previously unannotated lncRNA ORFs and 36 circRNA ORFs with coding potential. Among the linear candidates, lncORF32653 and lncORF98488 were proposed to encode 53- and 98-amino-acid products, respectively, and product expression promoted satellite-cell proliferation and differentiation. These experiments nominate functional coding events but do not establish whether the parent lncRNAs have independent RNA activities or whether endogenous myogenic phenotypes require translation of the nominated ORFs. The candidates therefore remain better classified as U than as established P- or B-type systems.

The unresolved candidates differ from the benchmarks primarily in experimental design, not chronology. Ribo-seq, reporter translation, and product overexpression can establish translation-associated evidence and product sufficiency, but they do not identify which molecular output causes an endogenous phenotype. Movement from U towards P requires selective loss of translation with preservation of the parent RNA, whereas a B designation additionally requires independent activity of the RNA output. Linc-RAM/MRLN therefore provides a system-level bifunctionality benchmark, SPAR and MTLN provide T4/F4 product-dependent regeneration benchmarks, and newer linear ORFs remain largely unresolved. This gradient provides the reference point for circRNA systems, where RNA-of-origin introduces an additional evidentiary bottleneck.

## 5. circRNAs: Translation Evidence Outpaces Causal Assignment

### 5.1. circZNF609: A Landmark Translation Claim Complicated by Source Attribution

circZNF609 is informative because substantial evidence exists for both circRNA function and translation, yet the causal link between them remains unresolved. Legnini et al. [72] identified circZNF609 as a conserved circRNA associated with skeletal muscle myogenesis and showed that its abundance decreases during myoblast differentiation. Depletion directed against the back-splice junction reduced proliferation of human primary myoblasts, whereas selective reduction of linear ZNF609 did not reproduce the same phenotype. These experiments established a biological requirement for the circular RNA species that could not be readily explained by loss of the linear host transcript. However, circRNA-specific depletion removes both the RNA molecule and any product translated from it. The resulting proliferation defect therefore establishes a circZNF609-dependent phenotype but does not identify the responsible molecular output. Under the functional-attribution axis defined above, this experiment remains F0 rather than evidence for an RNA-intrinsic function.

The original study also provided substantial evidence that circZNF609 is translation-competent. The circular transcript contains an open reading frame whose completion depends on back-splicing, and circZNF609 was detected in heavy polysome fractions with behaviour consistent with association with active translation machinery [72]. Engineered circZNF609 constructs produced FLAG-tagged products, while disruption of the relevant initiation codons abolished the corresponding signals, supporting translation competence. The study also went beyond ectopic expression by using endogenous genomic tagging. A CRISPR-engineered mouse embryonic stem-cell system introduced a FLAG tag into the endogenous locus, followed by immunoprecipitation and mass spectrometry, which identified a peptide consistent with the predicted circZNF609 product. Thus, the original evidence was not limited to a reporter assay. Even so, endogenous product support was comparatively limited and did not provide the multiple, source-discriminating peptide evidence that would now be required for high-confidence assignment of the protein to a circular template. Under the definitions used here, the endogenous genomic-tagging and mass-spectrometry result reaches reported T3-level evidence because it constitutes a direct endogenous product observation. The limited depth of that evidence, later failure to detect a robust endogenous product, and unresolved source attribution nevertheless reduce confidence; the assignment remains explicitly provisional and does not satisfy T4.

The source-attribution problem became clearer after systematic re-evaluation of circRNA translation constructs. Ho-Xuan et al. [41] showed that circRNA expression vectors can generate linear and aberrantly spliced RNA species capable of producing proteins initially attributed to the intended circular transcript. In the circZNF609 system, protein products could still be detected from modified constructs that were deficient in generating the expected circRNA, showing that protein production after vector transfection was not diagnostic of translation from the circular molecule itself. The study also failed to reproduce robust endogenous detection of the proposed circZNF609-derived protein. These observations do not formally exclude endogenous circZNF609 translation, particularly because the earlier endogenous genomic-tagging experiment represents a distinct line of evidence. They do, however, weaken the inference that proteins produced from circZNF609 expression constructs necessarily originate from the circular template. The case illustrates why product detection and RNA-of-origin assignment must be treated as separate questions. Circularity of the RNA, translation competence of the sequence, and existence of a protein product do not, individually or collectively, establish that the circular RNA itself was the template unless competing linear sources are adequately excluded.

Functional attribution remains unresolved as well. Neither the original study nor the subsequent re-evaluation directly tested whether circZNF609 translation is required for its effect on myoblast proliferation. A decisive experiment would compare wild-type circZNF609 with a translation-deficient circular transcript while preserving circularisation, abundance, and other relevant RNA properties, followed where possible by product rescue. Such output-specific experiments have not been reported for the original human/mouse myoblast system. The most defensible assignment is therefore reported/provisional T3 with F0 and unresolved (U): the T3 label records the original endogenous genomic-tagging/mass-spectrometry observation rather than a secure source-resolved product, and later reproducibility and RNA-of-origin concerns preclude T4 [41,72].

### 5.2. circFAM188B and circNEB: Why Product Evidence Must Be Graded by Endpoint

circFAM188B illustrates how translation competence and product activity can be established without resolving whether the phenotype attributed to the parent circRNA is translation-dependent. In chicken skeletal muscle satellite cells, circFAM188B promoted proliferation while suppressing myogenic differentiation, and a 103-amino-acid product was proposed to arise from its circular ORF [69]. Translation was supported by reporter assays and by expression of a FLAG-tagged circFAM188B construct. Mutation of the initiation codon abolished the tagged protein while retaining circRNA expression, showing that production of the 103-aa product depended on the nominated ORF. Mass spectrometry further identified peptides matching the predicted product in overexpression-based experiments. However, the two C-terminal amino acids that uniquely distinguish the circularly encoded sequence were not recovered by mass spectrometry. The product evidence therefore establishes experimental translation competence more securely than endogenous, source-specific protein production. Within the framework used here, circFAM188B is most conservatively placed at T2 rather than T3 or T4.

The functional experiments address a different question. Independent expression of circFAM188B-103aa promoted satellite-cell proliferation and suppressed differentiation, closely reproducing the direction of the phenotype observed after circFAM188B overexpression [69]. This concordance shows that the encoded product is biologically sufficient to influence the relevant myogenic processes. It does not establish that translation is necessary for the phenotype of the parent circRNA, nor does it demonstrate an autonomous RNA function. The study had already generated an initiation-codon mutant capable of separating circRNA expression from detectable protein production, but this construct was used mainly to validate translation rather than to perform a complete wild-type-versus-translation-deficient functional comparison across the myogenic phenotypes. No reciprocal product rescue was reported. The parallel effects of circFAM188B and circFAM188B-103aa are therefore compatible with a translation-dependent model but do not constitute evidence of bifunctionality. We classify this system as approximately T2/F1 and unresolved (U).

Unlike construct-only candidates, circNEB has direct evidence for an endogenous product. Huang et al. [68] combined RNA-seq, Ribo-seq, and peptidomics and proposed that a 907-aa, approximately 99-kDa product is generated by rolling-circle translation. Antibody detection in bovine muscle and fetal myoblasts supports endogenous product existence. Supplementary Figure S8 of Huang et al. [68] reports an SKP1 co-immunoprecipitation MS/MS spectrum assigned to a circNEB junction-encoded peptide; however, the displayed result does not by itself demonstrate unique endogenous circular-template provenance with alternative linear sources excluded. Extensive sequence overlap with canonical nebulin further limits shared-peptide and antibody discrimination. The assignment therefore remains T3 rather than T4, acknowledging both the junction-spectrum report and its source-attribution limits.

The circNEB functional assignment depends on the endpoint. Intact bos-circNEB overexpression or depletion altered bovine myoblast proliferation and fusion, and the in vivo injury experiments expressed the full parent circRNA rather than an isolated 907-aa product [68]. Supplementary Figures S9 and S10 of Huang et al. [68] similarly use the intact bos-circNEB expression construct for ubiquitination and injury-associated readouts. These principal phenotypes remain F0 because the intervention changes both the RNA and any translated protein. Product-directed co-immunoprecipitation and EGFP-peptide localisation/association experiments provide narrower protein-centred molecular evidence, which may be noted as F1-like only for directly reproduced product-only molecular readouts; they do not establish F1 sufficiency for ubiquitination, proliferation, fusion, or regeneration. We therefore report T3/F0/U for the main myogenic and regenerative endpoints and describe the product-association observations separately.

### 5.3. circSIK2: Moving from Product Sufficiency Towards Translation-Dependent Causality

circSIK2 combines endogenous product detection with a functional comparison using a translation-deficient circRNA, giving it greater causal resolution than many earlier translated circRNA candidates. Wang et al. [61] identified circSIK2 through an integrated circRNA-seq, Ribo-seq, and mass-spectrometry pipeline in chicken myoblasts and proposed that it encodes a 176-amino-acid product, SIK2-176aa. A custom SIK2-176aa antibody detected a signal in several chicken tissues, including breast and leg muscle, while liquid chromatography–tandem mass spectrometry (LC-MS/MS) of endogenous muscle samples recovered peptides covering substantial portions of the predicted product. The authors also noted that antibody specificity was better in DF-1 cells than in primary myoblasts or tissues. With LC-MS/MS carrying the greater weight, these data support endogenous product existence and a T3 assignment. The distinction from T4 remains important. Although the genome-wide screen identified several peptide spectra spanning circRNA back-splice junctions, the source-discriminating spectra reported for the screen were not shown to establish circSIK2 itself as the template of SIK2-176aa. Endogenous product existence is therefore considerably better supported than definitive RNA-of-origin attribution.

The study also examined how circSIK2 translation is initiated. Predicted internal ribosome entry site (IRES) elements did not produce convincing activity in the corresponding reporter assay, whereas N6-methyladenosine (m6A)-associated regulation provided an alternative mechanism [61]. METTL3 increased circSIK2 abundance and enhanced production of SIK2-176aa without comparably increasing linear SIK2 expression. The authors therefore proposed that m6A modification facilitates translation of the circular transcript. These data strengthen the biological plausibility of circSIK2 translation, although METTL3 affects both circSIK2 abundance and product output and therefore does not itself separate RNA-mediated from translation-dependent functions. The m6A experiments are therefore most informative on the translation axis rather than the functional-attribution axis.

The principal translation-selective comparison pairs the circSIK2 expression vector with a translation-deficient ATG-to-ACG variant [61]. Both express detectable circSIK2 without comparably altering linear SIK2, but the mutant lacks detectable SIK2-176aa production; its proliferation and myotube effects are attenuated relative to the wild-type circRNA construct. Independent SIK2-176aa expression supports product sufficiency. These matched construct-level data support F2 for the measured chicken-myoblast endpoints. Supplementary Figure S4 of Wang et al. [61] further shows that overexpressing the linear exon 7–9 fragment also raises the circSIK2 qRT-PCR signal, so it is not a strictly circle-free linear control. Because that figure reports transcript measurements but no matched protein-negative assay, it cannot independently resolve the endogenous RNA source.

These experiments do not fully separate RNA and product effects. Translation-deficient circSIK2 retained residual effects, which could reflect a separate RNA action, incomplete RNA-property preservation, or other construct-level differences. No endogenous circSIK2 translation-selective allele was tested in an injury-regeneration model, and no reciprocal matched product rescue was reported. SIK2-176aa expression in chicks increased muscle-fibre size, supporting in vivo product sufficiency for hypertrophy but not endogenous circRNA translation necessity or injury-induced regeneration. For the stated cultured-myoblast endpoints, circSIK2 therefore remains T3/F2/U: product contribution is supported, but the endogenous circular-template attribution remains unresolved.

### 5.4. Emerging Translated circRNAs: Growing Product Discovery Without Matched Causal Resolution

For emerging translated circRNAs, product-centred mechanistic studies often outpace matched tests of whether the parent-circRNA phenotype requires translation. circKANSL1L was reported to encode a 551-amino-acid protein that interacts with Akt and modulates FoxO3 activity during porcine myogenesis [85], and a chicken circZNF609 product was linked to proliferation and differentiation through IRES-like translation [86]. Tagged constructs, LC-MS/MS, and product-centred assays support biologically active products, but key experiments remain dominated by overexpression or do not compare a translation-deficient circular RNA with wild type for the principal phenotype. The limiting inference is output attribution, not the absence of a downstream mechanism.

A similar mismatch appears in translatome-scale discovery. Integrated RNA-seq and Ribo-seq in goat skeletal muscle satellite cells identified multiple lncRNA and circRNA ORFs, including circUSP25 and other products with activity after expression [84]. The same research programme subsequently identified circMAPK1-110aa, which interacts with MAP2K1 and reduces MAPK1/3 phosphorylation [87]. Pharmacological activation of MAPK signalling partially restored the affected cellular phenotypes, strengthening the pathway model. This is not, however, RNA-versus-product rescue: restoring a downstream pathway cannot determine whether the initiating phenotype arose from the circular RNA, its encoded product, or both. Without translation-selective disruption of circMAPK1 while preserving the circle, pathway depth should not be mistaken for output-specific causality.

In vivo product sufficiency can leave the same causal gap. circARHGAP10 was reported to encode ARHGAP10-202aa, supported by in vitro translation and immunodetection, and product-only expression promoted bovine myoblast differentiation [88]. In a cardiotoxin-induced mouse injury model, ARHGAP10-202aa increased MYL6 expression and accelerated regeneration, providing unusually direct evidence that the encoded product is biologically active in vivo. However, product-only expression remains a sufficiency experiment unless translation is selectively disrupted in the endogenous or parent circRNA and the corresponding phenotype is assessed. circARHGAP10 therefore remains F1 and unresolved with respect to dependence of the parent-circRNA phenotype on translation.

These systems are more informative when grouped by the missing inference than by gene name: product sufficiency without parent-RNA separation, pathway rescue without output rescue, and in vivo sufficiency without endogenous necessity. This grouping explains why product discovery and downstream mechanism can advance substantially while the causal identity of the responsible molecular output remains unresolved. circSIK2 remains methodologically distinct because translation was disabled within the parent circRNA and the resulting myogenic phenotype was tested directly.

Table 2 summarises the core endpoint-specific assignments and records the model and endpoint alongside each T/F state. Table 3 is restricted to boundary and emerging cases so that it does not duplicate the core classification.

**Table 2:** Endpoint-specific T × F classification of core translated ncRNA systems in skeletal muscle. * Reported/provisional T3, subject to later source and reproducibility concerns.

System	Model and Specified Endpoint	Decisive Experiment(s)	T × F/State	Remaining Uncertainty/Next Test
Linc-RAM/MRLN [36,70]	C2C12/mouse; Linc-RAM-dependent myogenic differentiation versus MRLN-dependent SERCA/Ca^2+^ handling.	MRLN-frame disruption preserves Linc-RAM pro-myogenic activity [70]; independent MRLN peptide experiments support SERCA-related function [36].	T3; F2 (RNA arm retained); Bsys (different endpoints)	No matched proof that both outputs mediate the same differentiation/regeneration endpoint; requires same-endpoint output-selective tests.
LINC00961–SPAR [62]	Mouse skeletal muscle; amino-acid-responsive mTORC1 signalling and cardiotoxin (CTX)-injury regeneration.	Endogenous SPAR start-site editing eliminates the product; tested LINC00961 RNA abundance is retained, with an in vivo injury phenotype [62].	T4; F4/P	Preservation beyond measured RNA abundance and same-endpoint product rescue remain untested; qualify output-specific inference.
MTLN/MPM/LEMP [63,71,79,80]	C2C12 differentiation; mouse myofibre and CTX-regeneration phenotypes (different contexts).	Lin et al. [63] used an endogenous 1-bp MPM frameshift with product loss, measured RNA abundance and an in vivo phenotype. The RNA-deleting LEMP mouse [71] is not an F4 test.	T4; F4/P (from [63])	447/811-nt RNA isoform contributions, unmeasured RNA properties, and matched in vivo product rescue remain unresolved.
circZNF609 [41,72]	T evidence from mouse embryonic stem cells; F endpoint from human primary-myoblast proliferation (different contexts).	Original endogenous 3 × FLAG/IP-MS yielded limited product evidence [72]; intact-circRNA knockdown affects human-myoblast proliferation; later reassessment disputes source specificity [41].	T3 * (reported/provisional); F0/U	No robust source-unique endogenous product or matched translation-deficient parent-circRNA phenotype in the relevant myoblast context.
circFAM188B [69]	Chicken primary skeletal-muscle satellite cells; proliferation and differentiation.	Tagged construct demonstrates coding capacity; independently expressed 103-aa product changes proliferation and differentiation [69].	T2; F1/U	WT versus translation-deficient parent circle was not phenotyped for the same endpoints; endogenous source not established.
circNEB [68]	T evidence from bovine muscle/fetal myoblasts; F endpoints from myoblasts and mouse/tree-shrew injury models.	Endogenous ~99-kDa antibody signal and reported SKP1-IP junction-associated spectrum; intact-circNEB manipulations underpin major myogenic/injury outcomes [68].	T3; F0/U (principal endpoints)	Circular-template uniqueness and RNA-versus-product dependence remain unresolved. Full-circRNA ubiquitination/injury tests are not product-only F1 tests.
circSIK2 [61]	Endogenous T evidence in chicken muscle; F2 in chicken primary myoblasts; separate chick hypertrophy sufficiency.	Tissue antibody/LC-MS/MS identifies SIK2-176aa; WT versus ATG-to-ACG circSIK2 constructs show altered myoblast phenotypes [61].	T3; F2/U (partial product contribution)	No circular-source-exclusive endogenous product or endogenous selective edit. Residual mutant effects and S4 linear-vector confounding remain.

Note: Assignments describe the indicated systems and endpoints and may combine T evidence from one tissue or cell context with F evidence from another, which is explicitly recorded in Supplementary Table S1; such a coordinate does not prove same-context product generation. Linc-RAM/MRLN is B at the system level because independent RNA and MRLN activities occur in different readouts, not because both outputs cause one regeneration endpoint. The circZNF609 T3 observation derives from endogenous tagging in mouse ES cells, whereas its F0 proliferation evidence is from human myoblasts; T3 is reported and provisional after methodological re-evaluation [41,72]. circNEB is F0 for principal proliferation, fusion, and injury phenotypes. Protein-localisation and association observations do not assign an F1 phenotype without product-only functional testing.

**Table 3:** Boundary and emerging translated ncRNA systems grouped by their unresolved causal inference.

System and Model/Endpoint	Decisive Translation/Source Evidence	Output-Specific Experiment and Endpoint	Interpretation/Status	Remaining Uncertainty/Next Test
lncRNA-Six1 [83]; DF-1 cells (primary functional assays); chicken myoblast observations	Expression-based ORF2 assay produced an approximately 7.26-kDa product [83].	ORF2 expression altered DF-1 proliferation, migration, and promoter readouts rather than a matched skeletal-myogenic phenotype.	Boundary; T2/F1-like, not core graded.	Require endogenous product and translation-selective perturbation in an appropriate myogenic/regenerative model.
Goat satellite-cell translated lncRNA/circRNA ORFs [84]; proliferation/differentiation	RNA-seq/Ribo-seq nominate translated ORFs; selected products are expressed experimentally.	Product expression changes satellite-cell phenotypes, without matched parent-RNA translation loss.	Boundary; U, not core graded.	Test endogenous product source and matched RNA-preserving translation loss for the stated endpoint.
circKANSL1L [85] (porcine) and chicken circZNF609 [86]; myogenic models	Tagged/product MS experiments support candidate encoded products, but source discrimination is incomplete.	Product-centred gain-of-function/pathway studies lack matched translation-deficient parent circles for principal phenotypes.	Emerging; U, not core graded.	Control alternative templates and repeat phenotyping after RNA-preserving translation loss.
circMAPK1 [87]; goat muscle satellite cells, proliferation/differentiation	circRNA/Ribo-seq evidence supports a candidate circMAPK1-110aa product.	Pharmacological MAPK pathway activation partially rescues cellular phenotypes; this represents pathway-level rescue rather than product-specific rescue.	Boundary; U, not core graded.	Require circMAPK1 translation-selective loss and matched encoded-product rescue, where feasible.
circARHGAP10 [88]; bovine myoblasts and mouse CTX injury	In vitro translation and immunodetection support a 202-aa product.	Independent product expression promotes myoblast differentiation and in vivo regeneration (sufficiency).	Boundary; F1/U for parent-circRNA dependence.	No RNA-preserving parent-circRNA translation loss for these endpoints; test endogenous provenance and necessity.

Note: These cases are included to illustrate recurrent inferential bottlenecks rather than to expand the core graded set. Product sufficiency, downstream-pathway rescue, or in vivo activity can establish biological relevance without proving that the parent-RNA phenotype depends on translation. T and/or F are therefore left unassigned where the available evidence was not formally core-graded. Figure 2 visualises the core systems on the two analytically distinct evidence dimensions and marks the decisive experiments still required for unresolved circRNA cases.

## 6. Discussion: From Translated-Product Discovery to Causal Output Maps

### 6.1. Why a Single Evidence Ladder Is Insufficient: Discordant T × F States

The T × F map is useful because it preserves evidence discordance rather than adding finer grades. Table 2 makes the limiting inference explicit for each core system, while Table 3 shows recurring bottlenecks in boundary cases. LINC00961-SPAR and MTLN combine source-resolved endogenous product evidence with endogenous coding-selective perturbations and retained measured parent-RNA abundance, meeting the operational T4/F4 criteria for defined regenerative phenotypes. circNEB has comparatively strong endogenous product evidence (T3) yet remains F0 for its principal proliferation, fusion, and regeneration phenotypes because intact circNEB was manipulated. circSIK2 occupies a different configuration at T3/F2 because translation was selectively disabled within the parent circle, but residual activity and the lack of an endogenous output-specific rescue leave complete separation unresolved. Among the core circRNA systems assessed here, none reaches both T4 source resolution and F4 endogenous output-specific causality. A single ordinal ladder would compress these biologically different configurations into a misleading sequence of ‘more’ or ‘less’ evidence even though the unresolved inference differs in each case.

These cases expose three common evidentiary substitutions. More convincing product detection cannot compensate for a missing output-discriminating functional experiment. A detailed downstream mechanism can explain how a product acts without showing that the parent transcript’s phenotype depends on that product. Likewise, an animal experiment does not automatically confer high functional attribution: product-only ARHGAP10-202aa expression in injured muscle establishes in vivo sufficiency [88], whereas SPAR and MTLN meet F4 criteria because endogenous coding-selective product-loss experiments test necessity with measured parent-RNA abundance retained [62,63]. The map is therefore diagnostic: it identifies which inference, rather than which molecule, remains weak. Figure 1 summarises these invalid substitutions. A 2026 chicken study combined RNA-seq and Ribo-seq to identify lncMPD, validated MPD-74aa expression by Western blotting and mass spectrometry, and showed that MPD-74aa overexpression promoted myoblast proliferation while inhibiting differentiation [89]. Because the functional tests centred on peptide gain-of-function rather than a matched translation-deficient lncMPD comparison, whether the parent-lncRNA phenotype depends on translation remains unresolved.

### 6.2. Evidence States Are Endpoint- and Context-Specific

R, P, B, and U are context-dependent rather than fixed properties of a locus. LINC00961 illustrates this directly. In skeletal-muscle regeneration, selective loss of SPAR without a significant change in measured host-RNA abundance supports a qualified product-dependent interpretation [62]. In endothelial cells, however, translation-deficient LINC00961 retains biological activity that differs from the SPAAR product [78]. The same transcript system can therefore occupy different output relationships across tissues and phenotypes without contradiction.

The B designation requires the same endpoint discipline. Linc-RAM/MRLN is experimentally bifunctional because translation-deficient Linc-RAM retains pro-myogenic activity while MRLN independently regulates SERCA-dependent Ca^2+^ handling [36,70]. This is system-level bifunctionality, not evidence that both outputs independently contribute to the same regeneration endpoint. Same-phenotype dual-output causality is a stronger claim and should require matched output-selective perturbation within that endpoint. Conversely, within the translated-ncRNA core set defined here, no system justifies an R-only label under the strict definition used. As an adjacent skeletal-muscle example, lnc-RMG regulates myogenesis through an RNA-mediated miR-133a-3p/MEIS2/TGFβR2 pathway, and lnc-RMG knockdown promoted skeletal-muscle regeneration [90]. Although this is not a translated-ncRNA case, it illustrates why an RNA-mediated arm should not be discounted when testing transcripts with coding potential.

circSIK2 illustrates why partially resolved systems should remain qualified. Residual activity after translation disruption could represent an autonomous RNA function, incomplete preservation of RNA properties, or unequal contributions of the two outputs across myogenic states [61]. A single endpoint or a single translation-deficient construct cannot distinguish these explanations. Every T/F assignment should therefore specify the biological state, endpoint, and model to which it applies.

### 6.3. RNA-of-Origin Is an Upstream Causal Constraint for circRNAs

For circRNAs, source attribution can become the limiting step before functional attribution is considered. In circZNF609, linear or aberrantly spliced species produced from expression constructs can generate proteins attributed to the intended circle [41,72]. circNEB presents a different problem: when a long predicted circular product overlaps extensively with a canonical host protein, shared peptides and antibody signals have limited power to identify the template [68]. RNA-of-origin is therefore more than a technical validation detail; it constrains whether a protein-mediated circRNA mechanism can be causally assigned at all.

In circRNA systems, T3 product detection cannot substitute for T4 source resolution, and functional evidence cannot repair unresolved template provenance. A product-mediated phenotype therefore cannot be assigned to endogenous circRNA translation while a plausible alternative linear template remains.

This source requirement creates a genuine technical asymmetry between circular and linear transcript systems. Shared exonic sequence can make source-unique endogenous peptides unavailable or difficult to detect, while perturbing the endogenous locus can affect circularisation and linear-transcript production together [40,41,64,65,66,67]. Achieving T4, and especially T4/F4, may therefore require source-discriminating and output-selective experiments that are technically more difficult than analogous tests in some linear systems. The absence of a T4/F4 circRNA among the core cases reviewed here should be read as the present evidentiary ceiling under common criteria, not as evidence that circRNAs are intrinsically less functional or that such causality is unattainable. Recent reviews summarise molecular mechanisms of circRNA translation [91,92], while a separate review synthesises circRNA-host gene biology in skeletal muscle myogenesis [93]. The former also highlights experimental artefacts from ectopic reporters and linear by-products [91]. Engineered circRNA systems can achieve highly efficient rolling-circle translation [94], but such construct-level efficiency demonstrates translation competence under engineered conditions rather than endogenous RNA-of-origin.

### 6.4. Experimental Priorities: Source Resolution, Output Resolution, and State-Conditioned Causal Mapping

Causal mapping requires three kinds of resolution. Source resolution asks which RNA template generated the endogenous product. Output resolution asks whether a defined phenotype persists when translation is selectively removed while the parent RNA is sufficiently preserved. State/endpoint resolution asks whether the relative contribution of RNA and product changes across satellite-cell activation, proliferation, differentiation, fusion, and injury-induced regeneration [1,2,3,13,14]. Each T × F coordinate should therefore be read as a context-specific causal snapshot rather than a permanent property of the locus.

Adding omics layers does not necessarily resolve the causal question. Translation-selective perturbations require direct measurement of parent-RNA abundance and, where relevant, circularisation, localisation, stability, and RNA–protein interactions [39,40]. Convergent results from independent translation-disabling alleles (for example, distinct start-codon and frameshift edits), RNA-property controls, and matched product rescue help address mutation-specific RNA effects; few single experiments eliminate every alternative. An endogenous back-splice-junction-specific peptide can support circRNA provenance only after sequence uniqueness, spectrum assignment, and plausible alternative templates have been assessed. Ribo-seq or polysome signals document ribosome engagement rather than a stable, source-resolved product, while ordinary mass-spectrometry peptides shared with a host protein do not resolve the RNA template. circRNA-selective depletion also requires linear-host-transcript controls, and overexpression constructs require explicit assessment of linear by-products [40,41]. Independent product expression or rescue addresses functional sufficiency, but should be matched to the phenotype and biological state. Reviews of lncRNA-derived microprotein validation summarise complementary ribosome profiling, proteomic, immunodetection, and endogenous-tagging approaches [95,96].

The cited systems have been studied in different myogenic states and models. circSIK2 was evaluated for proliferation and differentiation in cultured chicken myoblasts [61], whereas circNEB was examined for proliferation and fusion in bovine myoblasts and in separate injury models [68]. SPAR and MPM provide mouse injury/regeneration evidence [62,63], not matched measurements of both outputs at successive stages of one regenerative trajectory. Time-resolved output-selective perturbations are therefore needed to determine whether the RNA and product contributions change across stages.

Temporal transcriptomics, ribosome profiling, and targeted or data-independent acquisition (DIA) proteomics can show when each molecular output is present, but causal interpretation still requires perturbation. Single-cell or spatial approaches can help distinguish true state-dependent output switching from changes in cellular composition. Combined with stage-appropriate phenotyping, these measurements could turn descriptive translated-ncRNA catalogues into experimentally testable causal maps. A recent EMBO Journal review recommends convergent product validation and start-codon or frameshift controls when establishing cryptic-lncRNA translation, and separately notes the challenge of attributing physiological effects to the peptide rather than the host locus [97]. Large-scale translatome studies show extensive noncanonical translation and tissue- or cell-type-specific translation patterns [98,99], while proteomic/interactome analyses characterise interaction partners for evolutionarily young sORF products [100]. These resource-scale studies strengthen product discovery but do not, by themselves, resolve RNA-versus-product causality.

**Figure 3:**
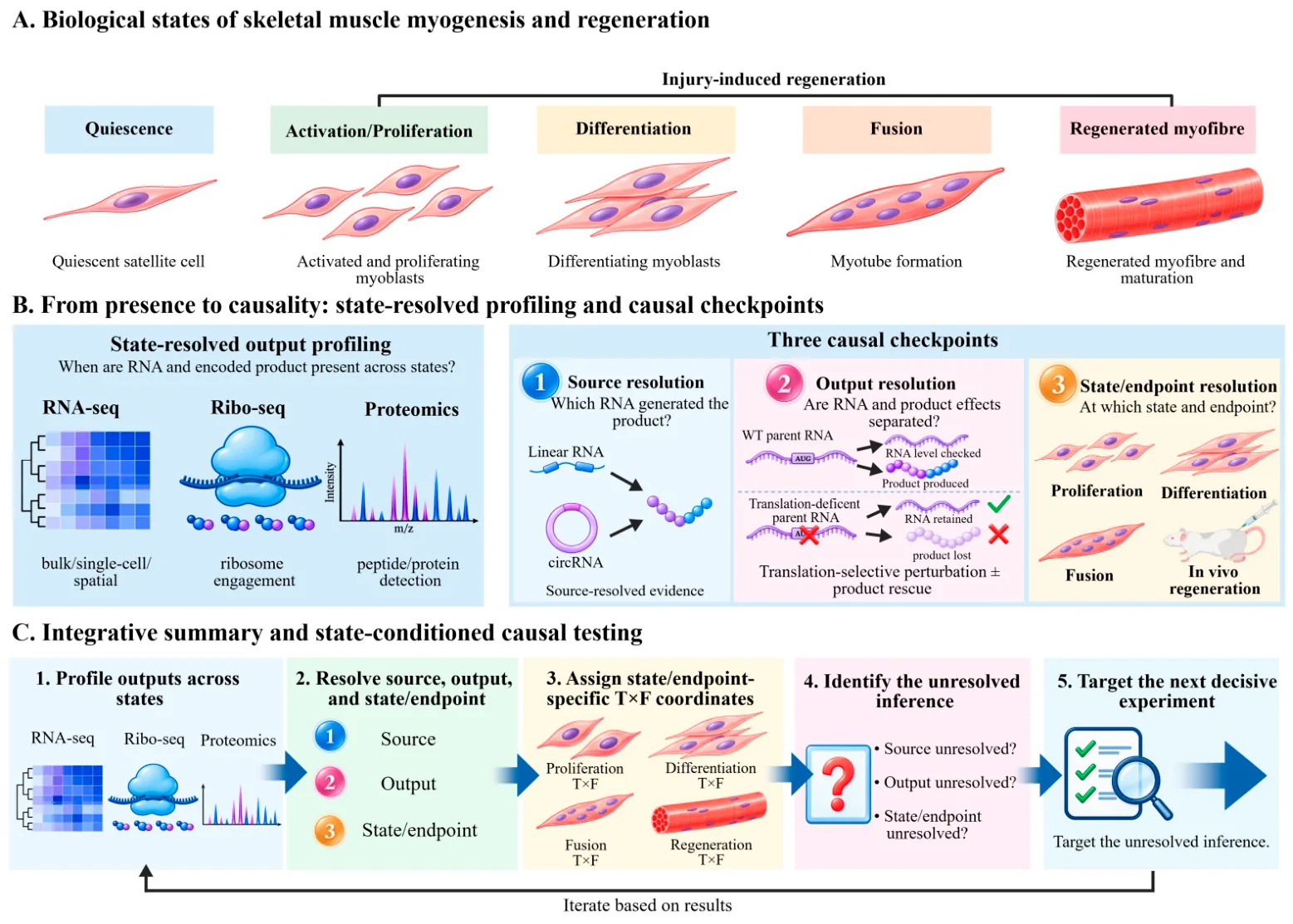
State- and endpoint-conditioned T × F workflow. (**A**) Quiescence, activation/proliferation, differentiation, fusion, and regenerated myofibre maturation are distinct skeletal-muscle states. (**B**) RNA-seq, Ribo-seq, and proteomics profile stage-dependent outputs; source, output, and state/endpoint resolution require complementary causal tests. Translation-deficient comparisons must verify product loss and retention of measured parent-RNA properties; RNA-abundance checks do not prove complete RNA equivalence. (**C**) The integrative summary assigns contextual T × F evidence states, identifies the unresolved inference, and selects the next decisive experiment. Coordinates cannot be transferred automatically across species or endpoints. Ribo-seq, ribosome profiling. Created with BioGDP.com [46].

The next experiment should target the unresolved inference rather than simply the next available assay. Systems at low T need better product/source evidence; systems with T3-level product evidence but low F need translation-selective functional tests; systems with partial output separation need endogenous output-specific perturbation, rescue where feasible, and state-resolved analysis. Figure 3 summarises this source–output–state workflow. The same causal logic can be applied beyond developmental myogenesis and acute injury, but the coordinates assigned here should not be transferred automatically to chronic disease contexts. circTmeff1 provides an atrophy-oriented proof of principle outside the regeneration-focused core [76], whereas ageing, muscular dystrophy, and other chronic myopathies would require new model- and endpoint-specific assignments using the same source- and output-resolution logic.

### 6.5. Framework Boundaries and Limitations

The T × F scheme classifies types of inference qualitatively; it is neither a validated ranking instrument nor a numerical study-quality score. Its numbered labels are not equally spaced, and F4 does not subsume F3 because endogenous necessity and matched product rescue answer different questions. The decision rules in Section 3 and the case-specific evidence record in Supplementary Table S1 make the assignments traceable but cannot eliminate all evaluator judgement, particularly near T3/T4 and F2/F3 boundaries. The assignments were independently assessed against operational criteria, with interpretive differences resolved by consensus; no formal inter-rater statistic is reported. Where source-specific or RNA-preservation evidence is incomplete, assignments remain qualified or provisional; the label cannot replace a missing control.

Coordinates remain conditional on species, cell state, intervention, product-detection assay, and specified endpoint. Evidence from C2C12 myoblasts, bovine fetal myoblasts, chicken satellite-cell cultures, and murine cardiotoxin-injury models cannot be treated as a single replicated causal experiment. Where T and F evidence come from different experimental contexts, a composite coordinate describes the evidence package rather than a same-sample test. The general decision rules can guide inquiry beyond skeletal myogenesis, but the empirical coordinates cannot automatically be transferred to human disease, ageing, or chronic atrophy. Translation-disabling edits may preserve measured RNA abundance while altering RNA structure, stability, localisation, circularisation, or binding [39,40]. Thus, “RNA-preserving” denotes only the properties tested, and representative sampling and the absence of a reported numerical agreement estimate limit claims about reproducibility and coverage.

Evolutionary conservation bears on cross-species portability but is not a third axis of causal evidence. Conservation should be considered at multiple levels, including locus synteny, RNA sequence or structure, ORF architecture, amino-acid sequence, and experimentally demonstrated output-specific function. SPAAR orthologs have been identified across mammalian lineages despite substantial primary-sequence divergence and evidence of conserved structural features [101]; LEMP has also been investigated in different vertebrate models [71]. Neither observation establishes an identical RNA-versus-peptide contribution in human, chicken, or bovine myogenesis. A recent review proposed that lncRNAs involved in ancient metabolic processes might face different evolutionary constraints from brain-associated lncRNAs, but presented this as a hypothesis requiring comparative testing rather than an established general rule [102]. Sequence conservation may guide candidate selection; T × F assignment still requires causal evidence specific to the model and endpoint.

## 7. Conclusions and Outlook

Translated ncRNAs in skeletal muscle do not fit neatly into either a coding-versus-non-coding dichotomy or a single evidence ladder. Product existence, RNA-of-origin, and output-specific causality are related but non-interchangeable inferences. The T × F map separates translation/source evidence from functional attribution so that ORF prediction, product detection, source attribution, product sufficiency, endogenous necessity, and bifunctionality are not collapsed into one ordinal claim. The core systems occupy different positions on this map. SPAR and MTLN meet the operational T4/F4 criteria for defined regenerative phenotypes, subject to the RNA properties measured and the limits of the published perturbations, while Linc-RAM/MRLN demonstrates separable RNA and micropeptide activities at the transcript-system level without proving same-phenotype dual causality. Among the core circRNA systems assessed here, product discovery is consistently better resolved than output-specific causality, and several retain a separate RNA-of-origin uncertainty. This pattern points to provenance and output attribution, rather than product discovery alone, as the principal bottlenecks limiting stronger causal claims in the current circRNA evidence base.

Progress will depend less on expanding the translated-ncRNA catalogue than on matching experiments to the inference that remains unresolved. Source-specific validation should establish which RNA generated the endogenous product; translation-selective perturbation with measured RNA-retention controls and product rescue or sufficiency testing should separate molecular outputs; and stage-appropriate phenotyping should determine when each output matters. Used this way, the T × F framework serves as a decision map for specifying which molecular output acts, in which biological state, on which skeletal-muscle phenotype, and what experiment is still required to justify that assignment.

## Figures and Tables

**Table 1:** Operational criteria and inferential boundaries of the T × F evidence framework.

Tier	Operational Criterion	What It Supports	Common Overclaim/What It Does Not Establish
T0	Computational ORF/coding prediction only	Candidate coding sequence	Neither translation nor an endogenous product
T1	Ribo-seq footprints or polysomes consistent with translation; specify specificity/periodicity if known	Translation-associated ribosome occupancy; for example, circSIK2 Ribo-seq [61]	Stable product, unique RNA source or phenotype
T2	Experimentally detected product from nominated sequence in a defined construct/reporter/in vitro system, with appropriate translation control	Translation competence; for example, circFAM188B construct assays [69]	Endogenous product or construct-free circular provenance
T3	Direct endogenous product signal with interpretable product-identity controls (MS, antibody or endogenous tag); mark disputed/limited results provisional	Endogenous product in sampled tissue; for example, SIK2-176aa muscle detection [61]	Specific RNA template where overlapping transcripts exist
T4	T3 plus source-discriminating endogenous evidence that addresses plausible alternative RNA templates (locus-selective edit, unique BSJ peptide or selective circRNA-to-product loss)	Source attribution at stated resolution; for example, SPAR/MPM locus edits [62,63]	Functional necessity, or automatic isoform resolution
F0	A specified RNA/locus phenotype from intact-transcript perturbation without RNA-versus-product separation	Transcript-system association; for example, intact-circNEB myogenic assays [68]	Identity of the causally responsible output
F1	Independently expressed product reproduces the same defined functional/molecular readout under suitable controls	Product sufficiency; for example, circFAM188B-103aa expression [69]	Requirement for translation of endogenous parent RNA
F2	Matched WT versus translation-deficient parent RNA in the same endpoint; target product loss and measured RNA retention. Report whether the RNA phenotype is lost, reduced, or retained	Attenuation supports product contribution (circSIK2 [61]); persistence supports a translation-independent RNA arm (Linc-RAM [70])	Full RNA functional equivalence; endogenous in vivo necessity; same-endpoint rescue.
F3	Same-endpoint matched product rescue in a translation-deficient RNA background, or controlled reciprocal RNA-only/product-only separation	Matched output-specific rescue/separation; no unequivocal F3 core benchmark is assigned here	Endogenous necessity if experiments are ectopic
F4	Endogenous coding-selective product loss with measured parent-RNA retention and corresponding in vivo phenotype; rescue not mandatory	Endogenous output-specific in vivo necessity; for example, SPAR/MPM edits [62,63]	All RNA functions preserved; obligatory F3 rescue; universal effect

Note: T and F are analytically non-interchangeable evidence dimensions rather than statistically independent variables or cumulative quality scores. Within the T axis, T3 and T4 are nested but conceptually distinct inferences: T3 establishes endogenous product existence, whereas T4 additionally requires source-specific attribution to the nominated RNA. T4 is therefore generally more demanding for circRNAs because linear transcripts can generate overlapping products. F states describe inferential reach rather than a mandatory cumulative checklist; an endogenous in vivo F4 necessity experiment can be decisive without reproducing every ectopic F3 rescue experiment. "RNA-preserving" is operational and refers only to the RNA properties that were actually measured; limited preservation checks should be reported explicitly and warrant qualification when the unmeasured properties could plausibly affect interpretation.

## Data Availability

No new experimental datasets were generated. The database-search strategy documentation, representative study-selection rationale, and evidence-adjudication rules are provided in the Supplementary Materials.

## Supplementary Materials

The following supporting information can be downloaded at: https://www.mdpi.com/article/10.3390/cells15211981/s1: Supplementary Table S1: Evidence matrix supporting endpoint-specific T × F assignments of translated non-coding RNA systems in skeletal myogenesis and regeneration; Supplementary Note S1: Operational protocol for T × F assignments; Supplementary Note S2: Original literature-search strategy and record documentation; Supplementary Table S2: Relation of the present T × F map to existing reviews and experimental standards; Supplementary Table S3: Names of representative RNA systems and encoded products.
