# Supplementary material for: From Translation to Causality: A Two-Axis Map of RNA-of-Origin and Output Attribution in Translated Non-Coding RNAs During Skeletal Muscle Myogenesis and Regeneration
Source: Cells. 2026 Oct 30;15(21):1981. doi: 10.3390/cells15211981 (PMC13762006; doi:10.3390/cells15211981)
Supplement: Supplementary file 1 [file cells-15-01981-s001.zip › cells-4640746-supplementary.pdf]

Supplementary Materials

Supplementary Table S1. Evidence matrix supporting endpoint-specific T × F assignments of translated non-coding RNA systems in skeletal myogenesis and regeneration.

Study / system	Model / defined endpoint	Translation / source evidence	T	Functional-attribution evidence / F state	Evidence strength, boundary and next decisive test
Linc-RAM/MRLN [36,70]	Mouse/C2C12; myogenic differentiation; independent SERCA/Ca2+ handling arm; cardiotoxin-injury evidence supportive.	MRLN is a 46-aa product from the Linc-RAM transcript system with endogenous product/physiology evidence. No source-resolved endogenous coding edit equivalent to T4 was reported.	T3	F2-type differentiation comparison: full-length MRLN-frameshift Linc-RAM retains differentiation-promoting activity rather than losing it, supporting a translation-independent RNA arm. MRLN independently regulates SERCA/Ca2+ physiology. System-level B, not same-phenotype dual-output causality.	Retained RNA myogenic activity after coding-frame disruption is the informative F2 outcome; attenuation is not a prerequisite of an F2 design. The separate peptide endpoint is Ca2+ handling. In vivo dual-output separation at the same endpoint remains untested.
LINC00961-SPAR [62]	Mouse; amino-acid-responsive mTORC1 signaling and acute-injury skeletal-muscle regeneration; host RNA checked in TA tissue.	Endogenous SPAR peptide detection plus start-site CRISPR allele with abolished product; genomic edit also altered a nearby SacII-recognition sequence. Endogenous source supported at LINC00961 locus (T4).	T4	F4/P for specified mouse injury phenotype. SPAR loss changes mTORC1 and regeneration while the measured LINC00961 qRT-PCR abundance is not significantly altered (Extended Data Fig. 7d, n=4). No complete same-endpoint endogenous product rescue.	Retained RNA property directly assessed: abundance, not global stability/structure/localization/RBP binding. Genetic editing changes RNA sequence. T4/F4 is operational and model/endpoint-specific; no claim that the mother RNA is universally inert.
MTLN/MPM/LEMP [63,71,80,81]	C2C12 differentiation; Lin et al. [63] mouse myofibre/CTX regeneration; Wang et al. [71] mouse development and injury use a distinct deletion.	Endogenous MPM/MTLN product detected; Lin et al. inserted 1 bp in the endogenous coding frame, terminating after aa5 and eliminating protein while measured host RNA persists. Two 447/811-nt isoforms share coding region; T4 at locus, not individually isoform-resolved.	T4	F4/P for Lin et al. [63] in vivo MPM-frame-mutant myofibre/CTX endpoints (skeletal-muscle qPCR WT/mutant n=5). Wang et al. [71] C2C12 CRISPR translation loss supports F2; its mouse whole-coding-region plus 3'UTR deletion lowers MyolncR4 RNA and does NOT independently qualify as F4. No matched in vivo peptide rescue.	Measured retention is steady-state RNA abundance, not equivalent RNA structure/localization/interaction. Lin product versus mutant RNA editing remains a possible cis-effect confound. Wang cell model and mouse deletion must not be pooled as identical output-selective interventions.
circZNF609 [41,72]	Product observation: mouse embryonic stem cells; proliferation F0 endpoint: human primary myoblasts (cross-context).	Endogenous genomic FLAG tagging/IP-MS yielded limited product evidence (reported T3). Ho-Xuan et al. [41] raised reproducibility and vector-derived linear-product concerns; circular-source specificity not confirmed.	T3*	BSJ-directed circZNF609 depletion reduces human-myoblast proliferation, but removes RNA plus any product. No translation-deficient circRNA phenotype in that cell context. F0/U.	Do not infer a same-context T3 product in human myoblasts from mouse-ES evidence. Preserve primary reported observation and subsequent counter-evidence. Need endogenous source-unique product and a matched RNA-preserving translation-loss experiment.
circFAM188B [69]	Chicken primary skeletal-muscle satellite cells; proliferation and differentiation.	Tagged/reporter translation competence and mutant-start-codon control; product MS from overexpression, but predicted circularly unique C-terminal residues were not recovered. T2 only.	T2	Independent expression of circFAM188B-103aa reproduces pro-proliferation/anti-differentiation readouts (F1). Translation-disabled parent-circle construct was not compared with WT for these same phenotypes; hence not F2.	Independently expressed product is sufficient under test conditions but parent-circRNA dependence remains unresolved. Next: WT/mutant circle matched phenotype, circRNA preservation controls, endogenous source-specific peptide.

Study / system	Model / defined endpoint	Translation / source evidence	T	Functional-attribution evidence / F state	Evidence strength, boundary and next decisive test
circNEB [68]	Endogenous T evidence: bovine skeletal muscle and fetal myoblasts; main F endpoints: myoblast proliferation/fusion and mouse/tree-shrew injury.	A ~99-kDa endogenous immunoreactive product plus proteomic analyses support T3. Figure S8 shows an SKP1 co-IP peptide spectrum assigned to a circNEB junction product, but no independent alternative-template exclusion; sequence overlaps canonical nebulin.	T3	F0/U for principal proliferation, fusion and injury outcomes because intact circNEB is altered. Fig. S9 ubiquitination and Fig. S10 tree-shrew injury use full-length bos-circNEB, not an isolated 907-aa protein. Product-specific binding/localisation is narrower molecular evidence and is not F1 for those phenotypes.	Source assignment remains incomplete despite reported Fig. S8. Product association may be noted as a narrow product-centred finding; do not label ubiquitination, proliferation or regeneration F1 without a product-only matched functional experiment. Need matched WT/translation-dead circle tests.
circSIK2 [61]	T evidence: endogenous chicken breast/leg muscle; F2 endpoint: cultured chicken myoblast proliferation and differentiation; chick hypertrophy via peptide-only delivery.	Endogenous SIK2-176aa antibody signal and muscle LC-MS/MS support T3; the five uniquely BSJ-crossing peptide spectra from initial screen do not individually identify circSIK2. Source-exclusive endogenous peptide evidence lacking.	T3	F2/U in chicken myoblasts: WT circSIK2 versus ATG-to-ACG circSIK2 construct attenuates but does not abolish the phenotype while measured circular RNA expression is retained. Separate chick product-only hypertrophy data show sufficiency, not endogenous F4. Residual effects do not independently establish RNA-only function.	Construct-level F2, not endogenous editing. S4 pCD3.1-Exon7-9 control raises the circSIK2 qRT-PCR signal and supplies no paired protein-negative panel. Residual mutant phenotype is not proof of RNA-intrinsic activity. Test endogenous source/provenance, RNA circularisation and RNA-property preservation, and matched rescue.
lncRNA-Six1 [84]	DF-1 cells for the principal proliferation, migration and promoter-activity assays; primary chicken myoblasts for localisation-related observations. Boundary case.	ORF2 generated an approximately 7.26-kDa product in an expression-based assay.	T2-like	ORF2-directed expression altered proliferation, migration and promoter activity, supporting product sufficiency in the tested context; the principal functional assays were outside the strict skeletal-myogenic core. F1-like; not core-graded.	Requires endogenous product evidence and RNA-preserving translation loss-of-function in a skeletal-myogenic or regenerative model before product-dependent or bifunctional assignment.
Translatome-derived ORFs [85]	Goat skeletal-muscle satellite cells during differentiation; includes lncORF32653, lncORF98488 and selected circRNA ORFs.	RNA-seq and Ribo-seq identified candidate translated ORFs; selected encoded products were expressed and showed biological activity.	NG	Selected product expression affected satellite-cell proliferation or differentiation, but the parent RNA and encoded product were not selectively separated. U.	Discovery and product sufficiency outpace causal attribution. Needs endogenous source confirmation and RNA-preserving translation loss-of-function before P or B assignment.
circKANSL1L [86]	Porcine myogenesis; product-centred cellular and pathway assays.	Tagged constructs, LC-MS/MS and product-centred experiments support a biologically active 551-aa product; source discrimination is incomplete in key experiments.	NG	The encoded product interacts with Akt and modulates FoxO3 during myogenesis, but the main phenotype lacks a matched translation-deficient parent-circRNA comparison. U.	Downstream mechanism does not establish that the parent-circle phenotype requires translation. Needs source-specific validation plus translation-deficient circKANSL1L with preserved RNA and matched phenotyping.
Chicken circZNF609 [87]	Chicken myoblasts; proliferation and differentiation.	An IRES-like region, tagged/product-centred assays and translation-focused experiments support a biologically active encoded product; source discrimination is incomplete in key experiments.	NG	Product-centred gain-of-function analyses show effects on proliferation and differentiation, but no matched translation-deficient parent-circRNA comparison establishes dependence of the circle phenotype. U.	Needs source-specific validation and RNA-preserving translation-selective testing of the parent circRNA. Product activity alone is insufficient for output-specific causality.
circMAPK1 [88]	Goat skeletal-muscle satellite cells; proliferation and differentiation.	circRNA-seq and ribosome profiling support a translated candidate; circMAPK1-110aa is linked to MAP2K1/MAPK1/3 signalling.	NG	Pharmacological MAPK activation partially restored cellular phenotypes, but this is downstream pathway rescue rather than RNA-versus-product rescue. U.	Requires selective disruption of circMAPK1 translation while preserving the circle, followed by matched phenotyping and product-specific rescue where feasible.

Study / system	Model / defined endpoint	Translation / source evidence	T	Functional-attribution evidence / F state	Evidence strength, boundary and next decisive test
circARHGAP10 [89]	Bovine myoblast differentiation; cardiotoxin-induced mouse muscle injury/regeneration.	In vitro translation and immunodetection support ARHGAP10-202aa production; source-resolved endogenous template evidence was not established in the core grading.	NG	Product-only ARHGAP10-202aa promoted myoblast differentiation and accelerated regeneration in vivo, establishing product sufficiency. F1 / U for dependence of the parent circle.	In vivo sufficiency is not endogenous translation necessity. Requires translation-selective loss-of-function of the parent circRNA with preserved RNA and assessment of the corresponding phenotype.

Note: T, translation/source evidence; F, functional attribution; R, RNA-intrinsic; P, product-dependent; B, experimentally bifunctional; U, unresolved; ORF, open reading frame; Ribo-seq, ribosome profiling; MS, mass spectrometry; LC-MS/MS, liquid chromatography-tandem mass spectrometry; BSJ, back-splice junction. T and F are analytically non-interchangeable, endpoint- and context-specific evidence dimensions, not quality scores. Within T, T3 denotes endogenous product existence, whereas T4 additionally requires source-resolved attribution to the nominated RNA. System-level B indicates independently demonstrated activity of both RNA and product and does not by itself establish same-phenotype dual-output causality. "RNA-preserving" refers only to properties actually measured; limited preservation checks warrant qualified interpretation when unmeasured properties could plausibly affect attribution. The first seven rows are core graded systems. NG, not formally graded; T3* denotes the reported/provisional circZNF609 T3 assignment, qualified by later reproducibility and RNA-of-origin concerns. Boundary/emerging rows remain ungraded where the manuscript does not make a formal T/F assignment. The matrix is representative rather than exhaustive.

Supplementary Note S1. Operational protocol for T × F assignments

1. Before assigning a coordinate, define the system, species, tissue/cell state, intervention and measured phenotype precisely. T- and F-evidence contexts are stated separately if they differ.

2. Assign T from directly observed product evidence and the ability to distinguish its RNA source. T2 is construct-level translation; T3 is direct endogenous product detection; T4 additionally excludes plausible alternative RNA templates. Provisional and locus-not-isoform-specific states are marked rather than silently rounded upward.

3. Determine F for the defined endpoint. F1 requires independent product expression reproducing that endpoint. F2 requires matched translation-competent versus translation-deficient parent RNA, intended product reduction/loss and measured RNA retention; record whether the phenotype is attenuated (supporting translation contribution) or retained (supporting a translation-independent RNA arm). F3 requires matched product rescue or reciprocal output-specific separation for that same endpoint. F4 requires endogenous coding-selective product loss, measured parent-RNA retention and a corresponding in vivo phenotype. F4 is a distinct route and does not require F3 rescue.

4. Record the measured RNA properties (abundance, relevant circularisation/BSJ, linear host, localization, stability, structure and binding where available), exact assay/figure, remaining source alternatives, and whether later work challenged a reported observation. Nonsignificant RNA-abundance differences are not formal equivalence tests.

5. Assign R, P, B or U as a separate interpretation. Product association or a residual phenotype after translation disruption does not alone establish an RNA-independent function; different output endpoints may justify system-level B without proving same-phenotype dual causality.

Boundary checklist (per model/endpoint; these categories are not a numerical score).

T2 to T3: Was the specified product directly detected in endogenous cells/tissue with adequate identity controls? If no, remain at T2. T3 to T4: Was its endogenous RNA template identified while plausible alternative transcripts were excluded? If no, remain at T3, marking challenged evidence as provisional.

F2: Were matched wild-type and translation-deficient parent RNAs compared for the same endpoint, with target-product loss and measured RNA retention? If no, do not infer F2 merely from a mutant construct. If yes, record whether the phenotype was attenuated or retained.

F3: Was the deficient phenotype rescued by the encoded product alone, or was equivalent reciprocal output-specific separation demonstrated for the same endpoint? Downstream pathway rescue alone is insufficient.

F4 (non-cumulative): Was the target product selectively lost by an endogenous coding edit while measured parent-RNA properties were retained and an in vivo phenotype changed? A whole-locus RNA deletion or product-only animal overexpression does not qualify; F3 rescue is not mandatory.

This protocol makes the authors' inferential decisions traceable. The T × F assignments were independently assessed against the predefined operational criteria. Differences in interpretation were resolved through discussion and consensus. These assignments represent qualitative, endpoint-specific evidence judgments rather than numerical quality scores or a formally validated rating instrument.

Supplementary Note S2. Original literature-search strategy and record documentation

The literature search was initially conducted in PubMed, Web of Science Core Collection and Scopus on 1 October 2026, with a publication cutoff of 30 September 2026. During revision, the authors recovered the original search strategies and associated records to complete this documentation. A targeted coverage check on 21 October 2026 supplemented the original records and informed minor refinements to the Boolean expressions presented below. The expressions below therefore incorporate the recovered search materials and supplementary revision-stage checks. This narrative review was not prospectively registered and does not constitute a systematic review.

Search concepts. Group 1 combined skeletal muscle, myogenesis, satellite-cell and regeneration terminology with translated lncRNA/circRNA, small-ORF and peptide terms. Group 2 traced named RNA/product systems discussed in the review. Group 3 captured product validation, source provenance and causal-attribution methods. Subject headings were used where available. The Boolean expressions reflect the recovered original strategies, with refinements used to check coverage during manuscript revision.

Web of Science Core Collection (Advanced Search)

W1 TS=(("skeletal muscle" OR "muscle regeneration" OR myogenes* OR myoblast* OR myotub* OR "satellite cell*" OR myofib*) AND (lncRNA* OR "long noncoding RNA*" OR "long non-coding RNA*" OR circRNA* OR "circular RNA*" OR "noncoding RNA*" OR "non-coding RNA*" OR micropeptid* OR microprotein* OR "small open reading frame*" OR sORF*) AND (translat* OR encod* OR peptid* OR microprotein* OR "ribosome profiling" OR "Ribo-seq" OR "open reading frame*" OR "mass spectrometr*"))

W2 TS=("Linc-RAM" OR MRLN OR LINC00961 OR SPAAR OR "SPAR polypeptide" OR MTLN OR "MPM micropeptide" OR LEMP OR MOXI OR mitoregulin OR circZNF609 OR circFAM188B OR circNEB OR circSIK2 OR circKANSL1L OR circMAPK1 OR circARHGAP10 OR "lncRNA-Six1" OR circTmeff1 OR lncMPD OR lncORF32653 OR lncORF98488)

W3 TS=((lncRNA* OR circRNA* OR "circular RNA*" OR "long noncoding RNA*" OR "long non-coding RNA*" OR micropeptid* OR microprotein*) AND (translat* OR "coding potential" OR "ribosome profiling" OR "small open reading frame*") AND ("evidence framework*" OR "evidence level*" OR "functional validation" OR "source attribution" OR "RNA-of-origin" OR "linear transcript*" OR "translation artifact*" OR "translation artefact*" OR "experimental validation"))

Scopus (Advanced Search)

S1 TITLE-ABS-KEY(("skeletal muscle" OR "muscle regeneration" OR myogenes* OR myoblast* OR myotub* OR "satellite cell*" OR myofib*) AND (lncRNA* OR "long noncoding RNA*" OR "long non-coding RNA*" OR circRNA* OR "circular RNA*" OR "noncoding RNA*" OR "non-coding RNA*" OR micropeptid* OR microprotein* OR "small open reading frame*" OR sORF*) AND (translat* OR encod* OR peptid* OR microprotein* OR "ribosome profiling" OR "Ribo-seq" OR "open reading frame*" OR "mass spectrometr*"))

S2 TITLE-ABS-KEY("Linc-RAM" OR MRLN OR LINC00961 OR "SPAR polypeptide" OR SPAAR OR MTLN OR (MPM AND (muscle OR micropeptid* OR mitochondri*)) OR (LEMP AND (micropeptid* OR myogenes* OR myoblast*)) OR (MOXI AND (micropeptid* OR "fatty acid oxidation" OR mitochondri*)) OR mitoregulin OR circZNF609 OR circFAM188B OR circNEB OR circSIK2 OR circKANSL1L OR circMAPK1 OR circARHGAP10 OR "lncRNA-Six1" OR circTmeff1 OR lncMPD OR lncORF32653 OR lncORF98488)

S3 TITLE-ABS-KEY((lncRNA* OR circRNA* OR "circular RNA*" OR "long noncoding RNA*" OR "long non-coding RNA*" OR micropeptid* OR microprotein*) AND (translat* OR "coding potential" OR "ribosome profiling" OR "small open reading frame*") AND ("evidence framework*" OR "evidence level*" OR "functional validation" OR "source attribution" OR "RNA-of-origin" OR "linear transcript*" OR "translation artifact*" OR "translation artefact*" OR "experimental validation"))

PubMed

P1 (("Muscle, Skeletal"[Mesh] OR "Satellite Cells, Skeletal Muscle"[Mesh] OR "skeletal muscle"[tiab] OR myogenes*[tiab] OR myoblast*[tiab] OR myotub*[tiab] OR "muscle regeneration"[tiab] OR "satellite cells"[tiab]) AND ("RNA, Long Noncoding"[Mesh] OR "RNA, Circular"[Mesh] OR lncRNA*[tiab] OR circRNA*[tiab] OR "circular RNA"[tiab] OR "long noncoding RNA"[tiab] OR "long non-coding RNA"[tiab] OR micropeptid*[tiab] OR microprotein*[tiab]) AND (translat*[tiab] OR encod*[tiab] OR "ribosome profiling"[tiab] OR "Ribo-seq"[tiab] OR "open reading frame"[tiab] OR "mass spectrometry"[tiab] OR micropeptid*[tiab] OR microprotein*[tiab])) AND 1900/01/01:2026/09/30[dp]

Record handling. Available exported records from the initial and supplementary searches were reviewed for source coverage and deduplicated by normalized DOI, with title matching for records without DOI. Distinct DOIs were not collapsed solely on title identity, preserving correction notices. Broad transcript-name queries can retrieve off-topic acronym matches; methodological and foundational papers were also found by citation tracing. This purposive narrative-review search did not entail exhaustive manual screening of every retrieved record or a PRISMA-style count of included studies. The seven core systems were selected to compare inferential questions, with criteria and limitations documented in Section 2 and Supplementary Table S1.

Supplementary Table S2. Relation of the present T × F map to existing reviews and experimental standards.

Previous approach	Established contribution	Distinct scope of this Review
Non-coding RNA and micropeptide reviews in skeletal muscle [42–44]	Organize RNA-mediated mechanisms, encoded products and muscle developmental or regenerative findings.	Map each specified muscle phenotype to separate product/source and functional-attribution evidence states.
CircRNA validation and source standards [40,41]	Specify circular-RNA identity checks, linear-template contamination concerns and product-validation controls.	Record source provenance as a T-axis constraint separately from F-axis RNA-versus-product causal testing.
Graded ncRNA-peptide evidence in cancer [45]	Uses ordered evidence from sequence/ribosomes through endogenous peptides and functional attribution for tumor settings.	Preserve high-T/low-F and lower-T/higher-F discordance without converting evidence types into one numerical rank.
Present skeletal-muscle T × F mapping	Integrates the established principles above; does not claim to invent their experiments.	Adds explicit endpoint/model context, four interpretive states (R/P/B/U), and next-experiment choices for seven representative muscle systems.

Supplementary Table S3. Names of representative RNA systems and encoded products.

System	Reported names	Usage and scope in this Review
LINC00961 / SPAR	LINC00961, SPAR, SPAAR	LINC00961 denotes the parent RNA; SPAR (also termed SPAAR) is its encoded product. RNA and product are not synonyms.
MTLN / MPM / LEMP	MTLN; MPM, LEMP, MOXI, mitoregulin; MyolncR4, 1500011K16Rik, LINC00116	MTLN is used as the common locus/product-system name; historical peptide and host-RNA labels are attributed to their original studies, with species/isoform context stated where relevant.
Linc-RAM / MRLN	Linc-RAM, MRLN (myoregulin)	Linc-RAM denotes the experimentally studied RNA output; MRLN denotes the encoded peptide. B_sys refers to independent activity of both outputs, not one shared phenotype.
circFAM188B	circFAM188B; circFAM188B-103aa	Parent circular RNA and candidate 103-aa encoded product are kept distinct.
circNEB	circNEB (bos-circNEB); circNEB-peptide	The proposed 907-aa product is not equated with the RNA, nor with the full-length canonical nebulin protein.
circSIK2	circSIK2; SIK2-176aa	circSIK2 denotes the parental circle; SIK2-176aa denotes its proposed translated product.
circZNF609	circZNF609; proposed circZNF609-derived product	Endogenous product evidence is reported/provisional; no particular peptide identity is treated as uniquely source-resolved.

Note: Alternative names do not demonstrate independent evidence of distinct products or functions. Preserve the species, RNA isoform, assay context and molecular output when comparing studies.
